# The value of percutaneous transhepatic treatment of biliary strictures following pediatric liver transplantation

**DOI:** 10.1590/0100-3984.2016.0087

**Published:** 2017

**Authors:** Leandro Cardarelli-Leite, Vinicius Adami Vayego Fornazari, Rogério Renato Peres, Alcides Augusto Salzedas-Neto, Adriano Miziara Gonzalez, Denis Szejnfeld, Suzan Menasce Goldman

**Affiliations:** 1 MD, Interventional Radiologist, Department of Diagnostic Imaging, Escola Paulista de Medicina da Universidade Federal de São Paulo (EPM-Unifesp), São Paulo, SP, Brazil.; 2 PhD, Interventional Radiologist, Department of Diagnostic Imaging, Escola Paulista de Medicina da Universidade Federal de São Paulo (EPM-Unifesp), São Paulo, SP, Brazil.; 3 MD, Surgeon, Department of Surgery, Escola Paulista de Medicina da Universidade Federal de São Paulo (EPM-Unifesp), São Paulo, SP, Brazil.; 4 PhD, Professor, Department of Surgery, Escola Paulista de Medicina da Universidade Federal de São Paulo (EPM-Unifesp), São Paulo, SP, Brazil.; 5 PhD, Professor, Department of Diagnostic Imaging, Escola Paulista de Medicina da Universidade Federal de São Paulo (EPM-Unifesp), São Paulo, SP, Brazil.

**Keywords:** Liver transplantation, Biliary atresia, Constriction, pathologic/therapy, Cholangiography, Drainage, Transplante de fígado, Atresia de vias biliares, Constrição patológica/terapia, Colangiografia, Drenagem

## Abstract

**Objective:**

To evaluate the percutaneous transhepatic approach to the treatment of biliary strictures in pediatric patients undergoing liver transplantation.

**Materials and Methods:**

This was a retrospective study of data obtained from the medical records, laboratory reports, and imaging examination reports of pediatric liver transplant recipients who underwent percutaneous transhepatic cholangiography, because of clinical suspicion of biliary strictures, between 1st September 2012 and 31 May 2015. Data were collected for 12 patients, 7 of whom were found to have biliary strictures.

**Results:**

In the 7 patients with biliary strictures, a total of 21 procedures were carried out: 2 patients (28.6%) underwent the procedure twice; 3 (42.8%) underwent the procedure three times; and 2 (28.6%) underwent the procedure four times. Therefore, the mean number of procedures per patient was 3 (range, 2–4), and the average interval between them was 2.9 months (range, 0.8–9.1 months). The drainage tube remained in place for a mean of 5.8 months (range, 3.1–12.6 months). One patient presented with a major complication, hemobilia, which was treated with endovascular embolization. Clinical success was achieved in all 7 patients, and the mean follow-up after drain removal was 15.4 months (range, 5.3–26.7 months).

**Conclusion:**

The percutaneous transhepatic approach to treating biliary strictures in pediatric liver transplant recipients proved safe, with high rates of technical and clinical success, as well as a low rate of complications.

## Abstract (pt)

**Objetivo:**

Demonstrar o valor da abordagem transparieto-hepática no tratamento de estenoses biliares em pacientes pediátricos submetidos a transplante de fígado.

**Materiais e Métodos:**

Estudo retrospectivo com revisão de prontuários, exames laboratoriais e de imagem dos pacientes pediá­tricos submetidos a transplante hepático. Foram incluídos pacientes com suspeita de estenose de vias biliares que realizaram colangiografia transparieto-hepática para diagnóstico, entre 1º de setembro de 2012 e 31 maio de 2015. Os dados de 12 pacientes foram coletados, dos quais 7 apresentaram estenose de vias biliares.

**Resultados:**

No total foram realizados 21 procedimentos: 2 pacientes realizaram dois procedimentos (28,6%), 3 pacientes realizaram três procedimentos (42,8%) e 2 pacientes realizaram quatro procedimentos (28,6%). A média de procedimentos por paciente foi 3 (variação: 2–4) e o intervalo médio entre os procedimentos foi 2,9 meses (variação: 0,8–9,1 meses). A permanência média do dreno foi 5,8 meses (variação: 3,1–12,6 meses). Uma paciente apresentou hemobilia com instabilidade hemodinâmica, tratada com sucesso por via endovascular. O sucesso clínico foi alcançado nos 7 pacientes e o seguimento médio após retirada do dreno foi 15,4 meses (variação: 5,3–26,7 meses).

**Conclusão:**

A abordagem transparieto-hepática das estenoses biliares em crianças submetidas a transplante de fígado demonstrou ser tratamento eficaz, com baixo índice de complicações.

## INTRODUCTION

The first attempt at pediatric liver transplantation was made in 1963 by the American surgeon Thomaz Earl Starzl in a 3-year-old boy with biliary atresia, who died during the surgical procedure. Since the development of new surgical techniques and immunosuppressive therapies in the 1980s, several groups of specialists in the United States, Europe, and Japan have each performed over five hundred pediatric liver transplantations, boasting a postoperative 10-year survival rate that exceeds 80%^(1)^.

Despite the ongoing improvement of surgical techniques and intensive care therapies, as well as the development of new immunosuppressive drugs, the procedure is by no means exempt from complications. As far as grafting is concerned, for example, the main obstacles are associated with arterial and biliary anastomoses^(2,3)^. Concurrently, biliary strictures are known to be the most common complications, occurring in up to 25% of pediatric liver transplantations^(4)^.

Clinically, biliary strictures should be suspected in patients presenting with cholestasis or episodes of cholangitis^(5)^. However, most patients present with a nonspecific clinical picture, together with discrete alterations in the levels of liver and bile canalicular enzymes^(6)^. When there is clinical suspicion of biliary stricture, noninvasive imaging tests can prove inconclusive. Abdominal ultrasound does not usually detect significant alterations, whereas magnetic resonance cholangiopancreatography, a tool superior to the former, presents an overall sensitivity of 50% in patients with biliary Roux-en-Y anastomosis, which is the most widely used technique in pediatric liver transplantation^(6-8)^.

Percutaneous transhepatic cholangiography has taken on a decisive role in diagnosing biliary stricture in pediatric liver transplant recipients inasmuch as it is considered the gold standard method for identifying and quantifying stenosis^(9)^. Percutaneous access also allows the treatment of these patients through discontinuous dilatation and drainage of the bile ducts. The goal of this study was to evaluate the percutaneous transhepatic approach in the treatment of biliary strictures in pediatric patients undergoing liver transplantation.

## MATERIALS AND METHODS

This was a retrospective study of data obtained from the medical records, laboratory reports, and imaging examination reports of pediatric liver transplant recipients who underwent percutaneous transhepatic cholangiography, because of clinical suspicion of biliary strictures, between 1st September 2012 and 31 May 2015, at a liver transplantation center. Patients in whom the test showed no alterations were excluded, as were those who did not remain in outpatient follow-up with the multidisciplinary team of the institution. The study was approved by the Research Ethics Committee of the Federal University of São Paulo Paulista School of Medicine, in the city of São Paulo, Brazil, and all patient data were kept confidential.

We collected clinical data for the 12 patients who underwent percutaneous transhepatic cholangiography upon suspicion of biliary stricture. Of those 12 patients, 7 tested positive for biliary stricture by cholangiography and were submitted to percutaneous biliary drainage on a successive dilatation schedule. Of those 7 patients, 4 (57.1%) were male, 5 (71.4%) presented with biliary atresia, 1 (14.3%) presented with metabolic disease, and 1 (14.3%) presented with autoimmune hepatitis. The mean age of those patients was 25.3 months (range, 6.0–105.5 months) at the time of transplantation and 59.3 months (range, 6.9–154.0 months) at the time of the first drainage procedure. Table 1 shows the characteristics of the patients submitted to biliary drainage.

**Table 1:** Profile of pediatric patients diagnosed with biliary strictures following liver transplantation and treated with percutaneous transhepatic drainage.

Patient	Gender	Underlying disease	Age at liver transplantation (months)	Age at first procedure (months)	Time from liver transplantation to stricture diagnosis (months)
1	Male	Metabolic disease	6.0	6.9	0.9
2	Male	Biliary atresia	25.1	36.9	11.8
3	Female	Autoimmune hepatitis	105.5	106.4	1.9
4	Female	Biliary atresia	9.6	57.3	47.7
5	Male	Biliary atresia	12.5	16.1	3.6
6	Female	Biliary atresia	10.4	22.1	11.7
7	Male	Biliary atresia	8.0	12.3	4.3

### Diagnostic parameters for assessing biliary strictures

**Figure 1:**
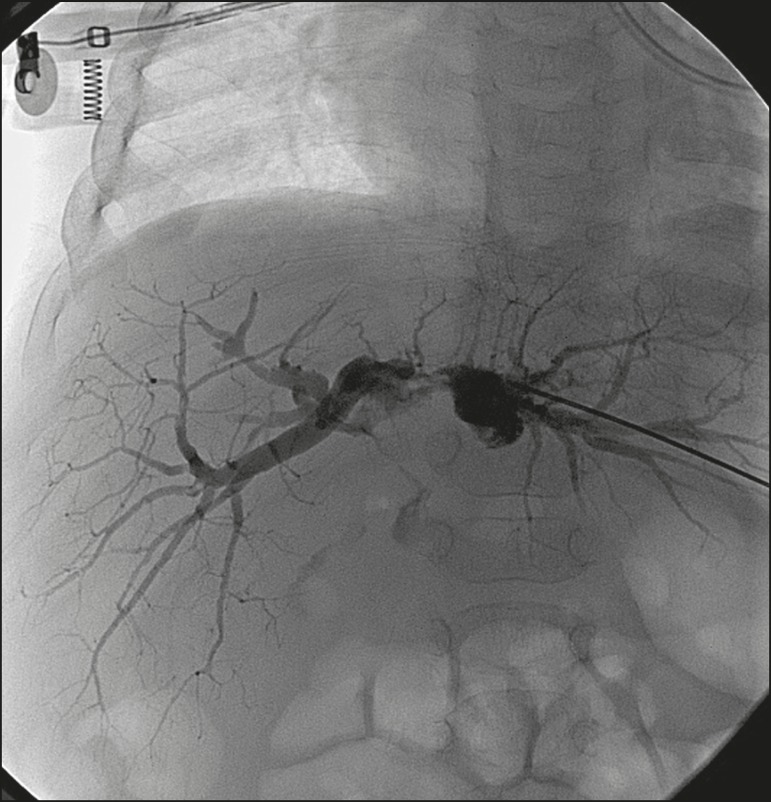
Cholangiography of a whole liver transplant recipient. Note the stricture in the biliodigestive anastomosis, promoting intrahepatic bile duct dilatation.

The diagnosis of biliary strictures was based on visualization of the dilatation of the intrahepatic bile ducts, with a sudden transition of caliber and a contrast medium outflow time greater than 3 min (Figure 1).

### Cholangiography and percutaneous transhepatic drainage

The procedures were carried out under general anesthesia. In the patients who were not undergoing antibiotic therapy, the prophylactic administration of second-generation cephalosporin was started immediately before the procedure and maintained for 7 days.

The procedures involved in gaining access to bile ducts and the initial diagnostic cholangiography were performed under direct fluoroscopic viewing (Integris V5000^®^; Philips Medical Systems, Eindhoven, the Netherlands) and employed a coaxial kit (NPAS 100^®^; Cook Medical, Bloomington, IN, USA) with a 22G Chiba needle (Cook Medical). For pediatric patients undergoing whole liver transplantation, the preference was for right-side puncture, on the midaxillary line, whereas left subxiphoid access was used in cases of partial transplantation. Abdominal ultrasound was used as an auxiliary method in the orientation of the biliary puncture. Low-osmolality nonionic iodinated contrast was administered in all cases.

Initially, 0.018” nitinol and 0.035” stiff hydrophilic guidewires were advanced through the bile ducts where transposition of the strictures occurred, 5F vertebral or multi-purpose catheters being employed, as necessary. Subsequently dilators ranging in size from 8F to 12F were advanced, and latex angioplasty balloon catheters (diameter, 6–8 mm; length, 20–40 mm) were inserted at the stricture point. The balloons were inflated, to the pressure recommended by the manufacturer, at least 3 times for approximately 60 s each. After dilatation, 8–12F internal-external drainage tubes were inserted, which were left open for 24 h after the procedure and then closed for patient discharge.

### Definition of technical and clinical success of biliary stricture treatment

Technical success was defined as transposition of the strictures, with posterior dilatation or balloon cholangioplasty and insertion of internal-external biliary drainage tubes. Clinical success was defined as symptomatic improvement, characterized by resolution of pruritus, jaundice, and cholangitis, together with normalization of biliary and liver enzymes.

### Treatment algorithm for patients presenting with biliary strictures

**Figure 2:**
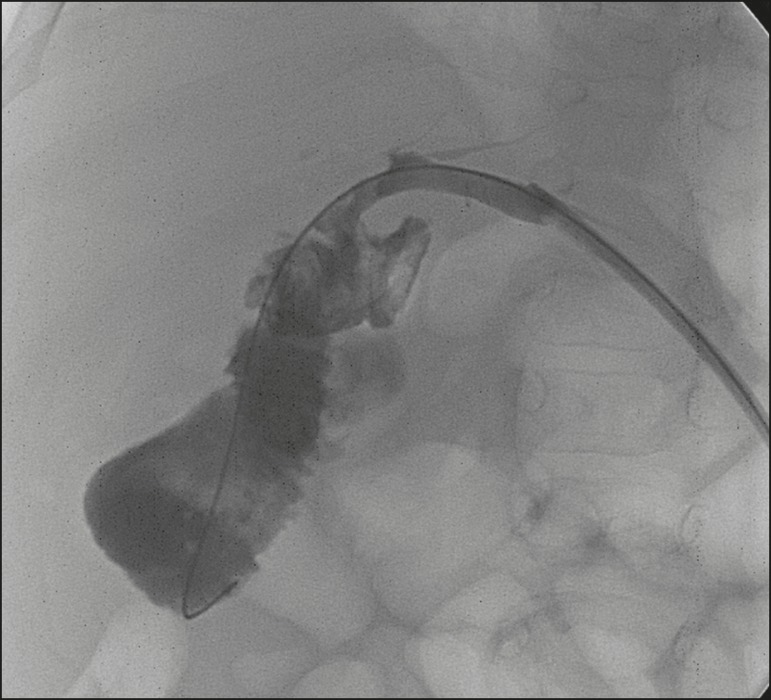
Cholangiography of the same whole liver transplant recipient depicted in Figure 1, after sequential cholangioplasty and biliary drainage. The contrast medium outflow time was less than 3 min, and there was no residual stenosis.

The treatment algorithm for patients presenting with biliary strictures consisted in carrying out percutaneous transhepatic cholangiography with the objective of diagnosing biliary stricture. After confirmation, duct dilatation as well as internal-external percutaneous biliary drainage was performed. After discharge, patients entered clinical follow-up, returning within two or three months for another percutaneous transhepatic cholangiography. In the case of resistant strictures, dilatation and drainage were again performed. If the transposition of the ducts was not viable in the first procedure, an external drain was left in place and a return visit for a new attempt was scheduled for within the month. When successful resolution of the biliary stricture was evident and clinical conditions had improved, the drain was removed and the patient remained under clinical follow-up (Figure 2).

## RESULTS

Among the 12 patients referred for an initial cholangiography, biliary stricture was confirmed in 7 (58.3%). Those 7 patients were submitted to transposition and dilatation of the strictures, together with internal-external drainage. Technical success of the first procedure was attained in 6 patients (85.7%), as shown in Table 2.

**Table 2:** Procedures performed per patient.

		First session				Second session				Third session				Fourth session		
Patient	Proc.	TPHC	Internal-external dilation and drainage	Interval (months)		TPHC	Internal-external dilation and drainage	Interval (months)		TPHC	Internal-external dilation and drainage	Interval (months)		TPHC	Internal-external dilation and drainage	Clinical success
1	2	Yes	Yes	3.1		Yes										Yes
2	3	Yes	Yes	3.3		Yes	Yes	4.9		Yes						Yes
3	3	Yes		0.9		Yes	Yes	2.3		Yes						Yes
4	4	Yes	Yes	2.1		Yes	Yes	1.4		Yes	Yes	9.1		Yes		yes
5	3	Yes	Yes	1.6		Yes	Yes	3.5		Yes						Yes
6	2	Yes	Yes	3.1		Yes										Yes
7	4	Yes	Yes	0.8		Yes	Yes	0.5		Yes	Yes	0.42		Yes		Yes

Proc., number of procedures carried out by patient; PTHC, percutaneous transhepatic cholangiography; Yes indicates that the step was carried out.

*Note:* a) Where the cell is blank, the variable does not apply; b) The “Interval” refers to the amount of time between the “Sessions” displayed on the left and on the right of its column.

In the 7 patients with biliary strictures, a total of 21 procedures were carried out: 2 patients (28.6%) underwent the procedure twice; 3 (42.8%) underwent the procedure three times; and 2 (28.6%) underwent the procedure four times. Therefore, the mean number of procedures per patient was 3 (range, 2–4), and the average interval between them was 2.9 months (range, 0.8–9.1 months). The mean time from liver transplantation to diagnosis of biliary stricture was 11.7 months (range, 0.9–47.7 months). The drainage tube remained in place for a mean of 5.8 months (range, 3.1–12.6 months). The mean follow-up after drain removal was 15.4 months (range, 5.3–26.7 months). One patient presented with a major complication—hemobilia after the second dilatation—which was treated with endovascular embolization. These results are summarized in Table 3. Sustained clinical success after drain removal was verified throughout the follow-up period in 100% of patients.

**Table 3:** Outcomes in pediatric patients diagnosed with biliary strictures following liver transplantation and treated with percutaneous transhepatic drainage.

Patient	Number of procedures	Mean time between procedures (months)	Mean drainage time (months)	Major complication after procedure	Follow-up period after drain removal (months)
1	2	3.1	3.1	-	18.3
2	3	4.1	8.2	-	11.1
3	3	1.6	3.3	Hemobilia	26.7
4	4	4.2	12.6	-	11.8
5	3	2.6	5.1	-	5.3
6	2	3.1	3.1	-	8.8
7	4	1.8	5.5	-	26.0

## DISCUSSION

Liver transplantation is currently the principal mode of treating end-stage liver disease. The rate of complications is higher in pediatric patients than in adult patients, because of the smaller calibers of the structures to be anastomosed in the former. The most common complications are those related to the liver itself. Biliary stricture should be suspected in patients presenting with cholangitis, jaundice, pruritus, and marked increases in biochemical markers of cholestasis^(10)^. However, the clinical-biochemical profile is often overlaid with other complications of a vascular, infectious, rejection-related, or graft dysfunction nature^(10)^. Noninvasive imaging tests are likely to produce a considerable number of false-negatives, and therefore the biliary stricture hypothesis cannot be ruled out even if abdominal ultrasound and magnetic resonance cholangiopancreatography reveal no alterations^(10)^. In addition, because most patients undergo biliodigestive anastomosis, which is mandatory in biliary stricture cases, the use of endoscopic retrograde cholangiopancreatography is less feasible^(3,11)^. Therefore, percutaneous transhepatic cholangiography has taken on added importance as a useful method of both diagnosing and enabling treatment of the stricture by means of dilatation and drainage during the same anesthesia session.

The first dilatation and drainage procedure usually poses the greatest challenge to interventional radiologists. Because the bile duct has not yet been approached, the degree of stricture is higher, there is more fibrotic tissue in the way, and the residual lumen is narrower, all of which renders the characterization and transposition more laborious. In addition, it is not unusual for a patient to present with cholangitis, which limits the volume of contrast injected into the bile ducts due to the risk of bacterial translocation and sepsis. In subsequent dilatations, when the patient already has the internal-external drain in place, procedures offer less complexity inasmuch as the course is secured and the stricture has already been transposed and dilated at least once.

In our case series, technical success was attained in 6 (85.7%) of the 7 initial procedures. In the remaining patient, it was not possible to transpose the stricture point and we therefore opted for external drainage in order to promote clinical recovery and lessen the local inflammatory process. After 28 days, another procedure was attempted and was successful. The 14 subsequent procedures were all successful. There was only one major complication, as previously described by Saad et al.^(12)^, namely an episode of hemobilia with hemodynamic instability, which was resolved through the use of hepatic arterial embolization, without the need for surgery^(13)^. Our experience is in consonance with data in the literature demonstrating the technical success of percutaneous transhepatic drainage applied to the treatment of pediatric biliary stricture and showing that it has a low rate of complications, which also included hemobilia in some studies^(5-8)^.

On average, each patients required three procedures, including the last one, in which the drain tube is removed and no dilatation occurs, with an mean interval of 2.9 months between each procedure. In comparison with those evaluated in other studies, our patients required a higher number of dilatations in order to achieve clinical success. In the studies conducted by Fonio et al.^(14)^ and Moreira et al.^(7)^, 60.0% and 65.7%, respectively, of the patients required only one dilatation. In the present study, however, this result was attained in only 2 (28.5%) of the 7 patients.

As for the periodicity of dilatation, there have been no randomized studies establishing the best interval. At our institution, we scheduled the procedure for once every two to three months, because we found that interval to be adequate for patient improvement, as well as because we took into account the limitations of some families, especially those residing in other states and who lack facility of transportation. In three procedures, the return visit was scheduled for more than three months after the previous procedure. That was due to an inability to establish contact with the family or to structural problems at the institution.

In the present study, the mean continuous drainage time required for complete resolution of the biliary stricture was 5.8 months. This parameter presents heterogeneous values in the literature, ranging from a little more than 30 days to as long as 20 months, indicating that there is no consensus among authors^(7,14)^. Inasmuch as we achieved clinical success in all 7 patients by the end of our study, this drainage time proved to be sufficient in our sample.

Patients who are younger at transplantation exhibit a higher biliary stricture rate, which is explained by the greater dimensions of the graft relative to the weight of the child, even when the split technique is used^(15)^. Nevertheless, long-term patency is greater in patients who underwent percutaneous biliary drainage before the age of three years^(16)^. In our study, patients presented with biliary stricture at an average age of 4 years and 11 months (59.3 months) and remained asymptomatic during an mean follow-up period of 15.4 months after drain removal.

If biliary stricture occurs within the first 30 days after pediatric liver transplantation, it must be assumed that there was a problem with the surgical technique^(3,15)^. In the present study, all patients presented with late biliary stricture, the diagnosis of which dated to nearly one year (mean, 11.7 months) after liver transplantation. Risk factors include recurring cholangitis, ABO incompatibility, chronic rejection, and cytomegalovirus infection^(17,18)^. Belenky et al.^(17)^ recommended that in the case of late biliary stricture, the option should be for a primary biliary stent placement, which shows long-term results superior to those obtained through isolated dilatation by drains or balloons. None of our patients underwent stent placement. In our routine, we avoid placing stents in children because the stents are prone to obstruction over time, which creates difficulty for those who will have to be submitted to a new surgical procedure in the future. The industry has recently introduced removable stent graft that can prevent the above-described problems. Despite their high cost, the use of such stents for the treatment of benign biliary strictures in pediatric patients merits further study^(19)^.

There have been no randomized studies comparing percutaneous biliary drainage and the surgical approach in terms of their success in resolving biliary strictures^(14,20)^. However, it is valid to suppose that surgery entails greater morbidity and risk, due to its longer duration, the need for longer periods of sedation, and the intense metabolic response to surgical trauma^(21)^. Our patients were initially treated with a minimally invasive percutaneous method and did not require open surgery for the resolution of their strictures. Therefore, we believe that interventional radiology has its place as an initial procedure for the management of biliary strictures in pediatric patients.

Our study has limitations related to its retrospective character and small number of patients. We were unable to obtain any information about the donor, graft cold ischemia time, the pretransplant clinical status of the recipient, or the technical report of all prior surgical procedures. In addition, the investigation that preceded the percutaneous biliary drainage approach did not follow the same protocol among recipients, because the heterogeneity of the clinical conditions of the patients was taken into account, as were the multifactorial causes that lead to biliary stricture.

## CONCLUSIONS

The percutaneous transhepatic approach to biliary strictures in children submitted to liver transplantation proved to be a safe treatment, with high rates of technical and clinical success, as well as a low rate of complications. In our case series, an average of three dilatations per patient, with an interval of three months between each, were required. The mean drainage time required for the resolution of the biliary stricture was 5.8 months.
